# Highlight: Virtual Issue on Host–Pathogen Interactions and Antimicrobial Drug Resistance

**DOI:** 10.1093/gbe/evac165

**Published:** 2022-12-08

**Authors:** Casey McGrath

A 2022 Virtual Issue from *Genome Biology and Evolution* (*GBE*) highlights recent articles on the evolutionary dynamics of host–pathogen interactions and antimicrobial drug resistance. This topic was the subject of a recent global symposium of the Society for Molecular Biology and Evolution (SMBE) organized by Santiago Castillo-Ramírez from the Universidad Nacional Autónoma de México, Ellen Leffler from the University of Utah, and Azim Ansari from the University of Oxford. This day-long symposium focused on coevolutionary relationships between pathogens and their hosts and the eco-evolutionary genomics of antimicrobial drug resistance. According to Leffler, the co-organizers “had been wanting to organize a symposium on this topic for a while, and even had one planned for SMBE 2020 [which was later canceled due to COVID-19]. We felt that host–pathogen studies were very exciting and in early stages, with a lot of creative research in diverse systems.” The global symposium, which was held on October 20, 2022, included 4 invited and 12 contributed talks, with the diverse group of speakers hailing from 4 continents. Following the symposium, *GBE* compiled this Virtual Issue of recent papers to foster further discussion and research on this topic.

Host–pathogen interactions and antibiotic resistance dynamics constitute good evolutionary models due to the strong selective pressures involved. As discussed by Jacques Fellay from École Polytechnique Fédérale de Lausanne in the first invited talk of the SMBE symposium, host–pathogen interactions leave signatures on the genomes of both organisms, allowing for interrogation of the coevolutionary forces at play. However, the specific genomic footprints observed may differ among host–pathogen systems due to differences in demographic histories, life-history traits, or genome architectures. For example, in their paper on the population genomics of the maize pathogen *Ustilago maydis*, Schweizer et al. (2021) observed lower-average genetic diversity compared with other fungal pathogens due to recent strong bottlenecks coinciding with maize domestication. They also found a higher rate of adaptive substitutions in genes located within virulence clusters, highlighting their role in *U. maydis* evolution. In contrast, in another plant fungal pathogen, the poplar rust fungus, *Melampsora larici-populina*, Persoons et al. (2022) found little impact of a selective sweep on overall genomic diversity outside of the immediate vicinity of the adaptive locus. This may indicate a “soft” selective sweep in which selection acted on standing variation and/or the impact of recombination in mitigating the loss of diversity.

Selective sweeps are not the only genomic signatures exhibited by pathogens adapting to their hosts. A study by Wyka et al. (2021) investigated the plant pathogen *Claviceps* and found that expansion of transposable elements and tandem gene duplications coincided with increased host range potential and speciation. According to Wyka et al., this indicates that “alterations of genomic architecture and plasticity can substantially influence and shape the evolutionary trajectory of fungal pathogens and their adaptability.” Consistent with this, Torres et al. (2021) identified polymorphic transposable elements in the fungal plant pathogen *Verticillium dahliae* that colocalized near pathogenicity-related genes and correlated with high expression levels, suggesting that these elements may impact the evolution of adaptive genomic regions.

Although these adaptive forces can increase pathogen virulence, they may come at a cost. A study by Cherry (2020) looked at gene inactivations driven by positive selection in the human pathogen *Salmonella enterica*. Cherry found that many of these genes were involved in virulence, motility and chemotaxis, biofilm formation, and resistance to antibiotics or toxins, providing a glimpse into the selective trade-offs often faced by pathogens in nature.

On the other side of this dynamic are the host genomes, which often exhibit their own unique signatures of host–pathogen interactions. Liu et al. (2020) explored these host genomic signatures by investigating the evolutionary history of the toll-like receptor (TLR) gene family across vertebrates. The authors found evidence for positive selection acting on codons in ligand-binding extracellular domains, suggesting the influence of pathogens in driving host TLR evolution.

In her invited talk at the SMBE symposium, Kayla King from the University of Oxford discussed ways in which additional symbioses can impact host–pathogen dynamics, either by affecting the host’s susceptibility to pathogens or by actively promoting host defense. As an example of the first type, Fiedoruk et al. (2020) investigated the evolutionary history of *Pseudomonas aeruginosa* filamentous bacteriophages, which contribute to the pathogenicity of this opportunistic bacterium. The authors revealed the presence of two evolutionary lineages, only one of which has been previously studied in terms of its role in pathogenesis. Filamentous bacteriophages were also studied in *Neisseria* by Al Suwayyid et al. (2020). Although the meningococcal disease–associated phage had been previously found to be associated with strains of *Neisseria meningitidis* that cause invasive meningococcal disease, Al Suwayyid et al. discovered its presence in an isolate of *Neisseria gonorrhoeae* causing gonococcal meningitis, as well as in the commensal species, *Neisseria lactamica* and *Neisseria cinerea*. Additional studies are needed to determine how this phage may affect colonization in these other species.

As an example of microbe-mediated host defense, a recent study by Dyrhage et al. (2022) analyzed the genomes of *Apilactobacillus kunkeei* isolates obtained from the honey crop (foregut) of honeybees and found that a subset of these strains produced the antimicrobial compound kunkecin A and inhibited growth of the bee pathogen *Melissococcus plutonius*. Thus, *A. kunkeei* plays a key role in honeybee pathogen defense, adding new complexity to traditional host–pathogen dynamics.

Antimicrobial resistance represents another facet of host–pathogen interactions and the evolutionary arms races that often result. At the SMBE symposium, invited speaker Vaughn Cooper from the University of Pittsburgh discussed how the environmental structure and ecological interactions promoted by bacterial biofilms can alter the evolutionary dynamics of antibiotic-based selection and the development of antibiotic resistance. Consistent with this, in laboratory-based evolution experiments with *P. aeruginosa*—an opportunistic pathogen affecting those with cystic fibrosis—Schick et al. (2022) found that reduced dispersal in spatially structured populations ultimately led to a reduction in driver mutations and more variable evolutionary trajectories. Interestingly, they also found that several mutations commonly observed in cystic fibrosis patients could be explained by adaptation to a novel environment alone and were not necessarily induced by antimicrobial treatment or immune system interactions.

In the final invited talk at the SMBE symposium, Amy Cain from Macquarie University discussed the current dearth of new antibiotics in the development pipeline and emerging methods for identifying new potential drug targets. Another potential avenue for exploration is investigating the origin and evolution of naturally occurring antibiotic compounds. Li et al. (2020) investigated the evolution of biosynthetic pathways for fusidane-type antibiotics across fungal genomes and found evidence for multiple horizontal gene transfer events between distinct classes of fungi. It is also critical to gain a better understanding of the evolutionary histories of antibiotic-resistant pathogens. To this end, Joseph et al. (2022) tracked the emergence and global dissemination of a *N. gonorrhoeae* lineage with increased resistance to azithromycin, revealing the key role of recombination and selective pressures in establishing this successful gonococcal lineage. Finally, Alama-Bermejo et al. (2020) developed a new bioinformatic pipeline for studying *Ceratonova shasta*, a pathogen of salmonid fishes. In the past, host genome contamination has been a major barrier to genomic analysis of this pathogen. The new method developed by Alama-Bermejo et al. allowed them to identify virulence-associated genes related to motility and proteolysis that may be good targets for future prevention and control efforts.

Together, the articles presented in *GBE*’s Virtual Issue on Host–Pathogen Interactions and Antimicrobial Drug Resistance support and reinforce many of the talks and discussions held at the SMBE 2022 global symposium. With this collection, *GBE* aims to promote additional research and provide new insight into this field, a sentiment that is shared by symposium co-organizer Leffler: “I hope dual host–pathogen studies will expand to more pathogens and larger samples, as it is a powerful approach and may teach us a lot about the effect of these strong selective pressures.”
